# Genome-Wide Identification of the *Salvia miltiorrhiza* *SmCIPK* Gene Family and Revealing the Salt Resistance Characteristic of *SmCIPK13*

**DOI:** 10.3390/ijms23126861

**Published:** 2022-06-20

**Authors:** Shuang Wang, Qi Li

**Affiliations:** College of Life Science, Sichuan Agricultural University, No. 46, Xinkang Road, Ya’an 625014, China; lq760621985@gmail.com

**Keywords:** *CIPK* gene family, transcriptome analysis, salt stress, *Salvia miltiorrhiza*, transgenic *Arabidopsis*

## Abstract

Members of the CIPK (CBL-interacting protein kinases) gene family play important roles in calcium (Ca^2+^) signaling pathway-regulated plant resistance to abiotic stresses. *Salvia miltiorrhiza*, which is widely planted and grown in complex and diverse environments, is mainly focused on the transcriptional regulation of enzyme genes related to the biosynthesis of its bioactive components. However, the excavation of the genes related to the resistance of *S.miltiorrhiza* and the involved signaling pathways have not been deeply studied. In this study, 20 *SmCIPK* genes were identified and classified into two families and five subfamilies by biochemical means. Sequence characteristics and conserved motif analysis revealed the conservation and difference of SmCIPK protein in plants. Expression pattern analysis showed that *SmCIPKs* were mainly expressed in flowers and roots, and more than 90% of gene expression was induced by SA (salicylic acid), and MeJA (methyl jasmonate). Furthermore, the expression level of *SmCIPK13* could be significantly increased after stress treatment with NaCl. *SmCIPK13* expression in yeast reduces sensitivity to salt, while overexpression of it in *Arabidopsis* has the same effect and was localized in the cytoplasm, cell membrane and nucleus. In conclusion, the identification of the *SmCIPK* gene family and the functional characterization of the *SmCIPK13* gene provides the basis for clarification of key genes in the Ca^2+^ signaling pathway and abiotic stress in *S.miltiorrhiza*.

## 1. Introduction

In plant cells, Ca^2+^ is recognized as a major messenger involved in the regulation of a wide range of plant stress responses to adversity and the formation and development of plant tissues and organs. Previous studies have shown that a variety of abiotic and biotic stresses can cause rapid changes in intracellular Ca^2+^ in plants [1,2]. Plants adapt to various adversity environments by sensing intracellular ion changes and thereby generating specific physiological and biochemical responses. CBLs, as Ca^2+^-dependent receptors, are not enzymatically active and are activated after sensing Ca^2+^ signals and interacting with their downstream target proteins, CIPKs, which form the CBL-CIPK signaling system, leading to a downstream cascade response [3,4].

The function of the Ca^2+^ receptor CBL family involved in signal transduction in vivo is mainly dependent on the CIPK pathway, which regulates downstream target genes through the CBL-CIPK signaling pathway [5]. CBL-CIPK plays an important role in the signal transduction process of plants in response to salt stress. In the model plant *Arabidopsis thaliana*, it was found that CBL1, CBL4, CBL5 and CBL10 play key roles in response to salt stress. There are two major salt stress signaling pathways: the CBL4-CIPK24-SOS1 (salt overly sensitive 1) pathway, which was one of the first pathways studied in the CBL-CIPK signaling pathway and is also known as the SOS (salt overly sensitive) pathway, and the other is the CBL10-CPK24-NHX (Na^+^/H^+^ antiporter) pathway [6,7]. The former is mainly to improve the tolerance of plants to salty environments by excreting excess Na^+^ from cells; the latter is mainly to excrete excess Na^+^ from cytoplasm to vesicles to maintain the normal state of Na^+^ in cytoplasm. In addition, it has been found that changes in SA concentration activate the Ca^2+^ signaling pathway by salicyl hydroxamic acid (SHAM)-sensitive peroxidases-mediated ROS (reactive oxygen species) production [8].

CIPKs are derived from the SnRKs family of the CDPK-SnRK (calcium-dependent protein kinase/Snf1-related protein kinase) superfamily and belong to the plant-specific serine-threonine protein kinases [9,10]. To date, 26 CIPKs have been identified in *Arabidopsis thaliana* and 30 in rice [11,12]. CIPK proteins have an N-terminal kinase domain and a C-terminal regulatory domain, and an activation loop within the N-terminal kinase catalytic domain is located between the conserved DFG and APE-motif [13,14]. Protein kinases are activated by phosphorylation of one or more amino acid residues in the activation loop [15], which are phosphorylated by the presence of free hydroxyl groups, mainly on Y (tyrosine), S (serine) or T (threonine) residues. In *Arabidopsis thaliana*, three sites, S, T and Y, are conserved in the activation loop of all 26 CIPKs [13,16]. The C-terminal regulatory region has a highly conserved NAF domain with a highly conserved N-A-F (asparagine-alanine-phenylalanine) consisting of 24 amino acids [17], which is also known as the FISL motif because of the absolute conservation of amino acids at the A, F, I, S, and L sites in this region [13]. Under normal conditions, the activity of CIPKs is inhibited by the intramolecular binding of NAF and CIPK catalytic domains, and after being subjected to signaling the NAF-motif is used to bind CBLs and thus CIPKs are activated, which may lead to specific recognition of CBLs and CIPKs due to varying degrees of variation in their sequences [18]. Currently, a relatively conserved PPI (protein phosphatase interaction) motif of 37 amino acids adjacent to the right side of the NAF-motif is capable of protein interactions with the PP2Cs family (protein phosphatase 2C) [19].

Tanshinone and phenolic acids are the most important active substances of *S.miltiorrhiza*, and their transcriptional regulatory mechanisms are well understood, However, in the process of field cultivation and production, the growth and development of *S. miltiorrhiza* and seed germination are both greatly affected by soil salinity. In this study, 20 *SmCIPKs* were predicted and identified in the *S. miltiorrhiza* genome and analyzed for phylogenetic classification, gene structure, conserved motif and expression pattern. *SmCIPK13*, which was expressed at relatively high levels in root, stem and leaves and induced by SA and NaCl, was selected as a candidate gene for functional exploration. Subsequently, *SmCIPK13* expression was found to enhance resistance to salt stress in yeast and transgenic *Arabidopsis thaliana* and to be localized in the cytoplasm, cell membrane and nucleus. As a result, our results provide a reference for breeding highly salt-resistant *S. miltiorrhiza* species by molecular means.

## 2. Result

### 2.1. Determination and Sequence Characterization of SmCIPKs Gene Family Members

In total, 20 SmCIPKs were obtained by screening the *S. miltiorrhiza* genome using Pfam, HMMscan, and SMART. The length of amino acids of *SmCIPKs* genes ranged from 375 (SmCIPK7) to 507 (SmCIPK16) (Table 1). The lowest and highest isoelectric points were 5.52 (SmCIPK16) and 9.56 (SmCIPK15), respectively, with 70% exhibiting acidity. The average molecular weight of SmCIPKs protein was about 49.3 kDa. The predicted results showed that SmCIPK5/8 was localized in cytoplasm and nucleus, SmCIPK9/11/16 in centrosome, cytoplasm and nucleus, and the remaining members were localized in cytoplasm.

### 2.2. Phylogenetic and Sequence Analysis of SmCIPKs

**Figure 1:**
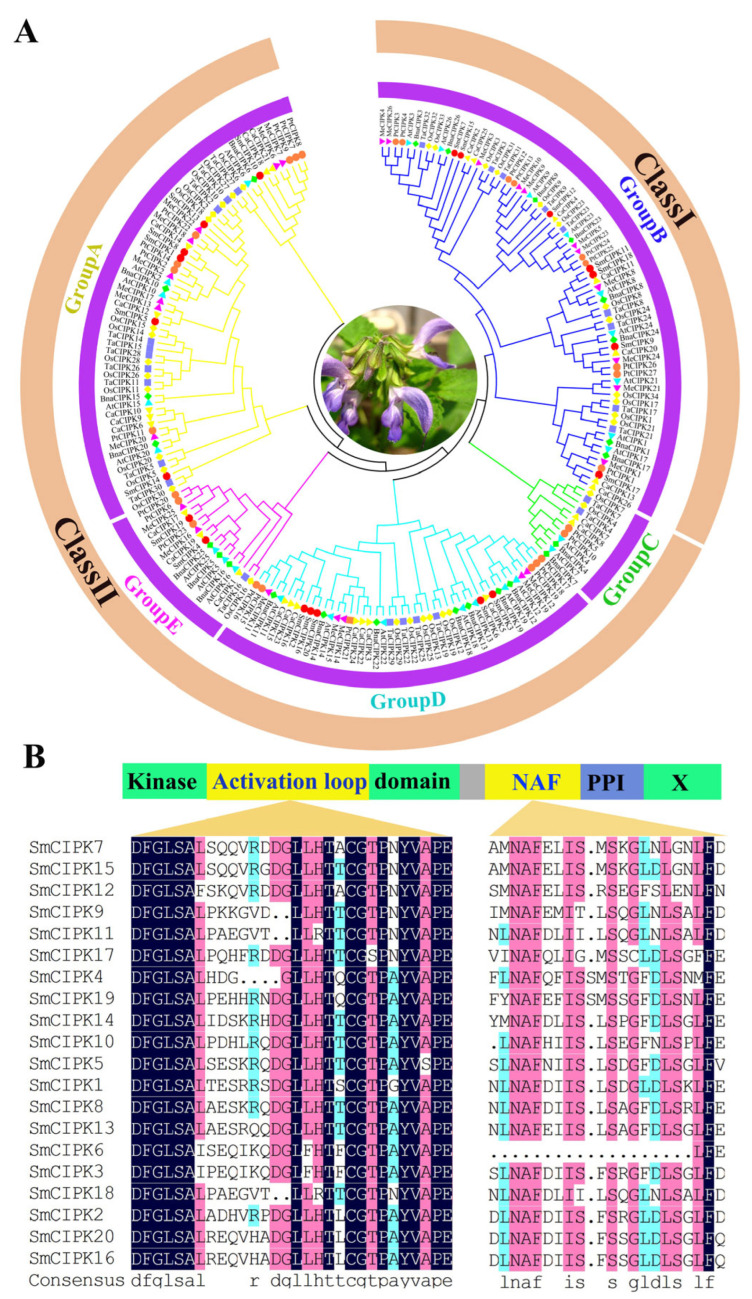
Phylogenetic analysis and sequence alignment analysis of SmCIPKs. (**A**). Phylogenetic trees of SmCIPKs were established with sequences of CIPKs in *Capsicum annuum*, *Arabidopsis thaliana*, *Manihot esculenta*, *Populus trichocarpa*, *Oryza sativa*, *Triticum aestivum*, and *Brassica napus* species. (**B**). The SmCIPKs protein major activation loop sequences and the corresponding sequences of the proteins within the conserved structural domain of NAF were compared.

To clarify the phylogenetic relationships between *S. miltiorrhiza* CIPKs and CIPKs in other species (*Capsicum annuum*, *Arabidopsis thaliana*, *Manihot esculenta*, *Populus trichocarpa*, *Oryza sativa*, *Triticum aestivum*, and *Brassica napus*), an evolutionary tree of 182 CIPK members was built (Figure 1A). The results show that there are 2 clades: clade I containing 82 members and clade II containing 100 members, of which clade II can be divided into 4 subclades: A, C, D and E. The amino acid sequence alignment revealed that SmCIPKs have two structural domains: a conserved N-terminal kinase structural domain and a C-terminal regulatory structural domain (Figure 1B). The N-terminal kinase catalytic domain has an activation loop located between the conserved DFG and APE-motifs and contains conserved phosphorylatable primary sites on Y, S or T residues. The N-A-F is highly conserved and consists of 24 amino acids in the NAF domain, due to the absolute conservation of amino acids at the A, F, I, S and L sites in this region. Unexpectedly, the NAF domain of SmCIPK6 is incomplete compared to other SmCIPKs.

**Figure 2:**
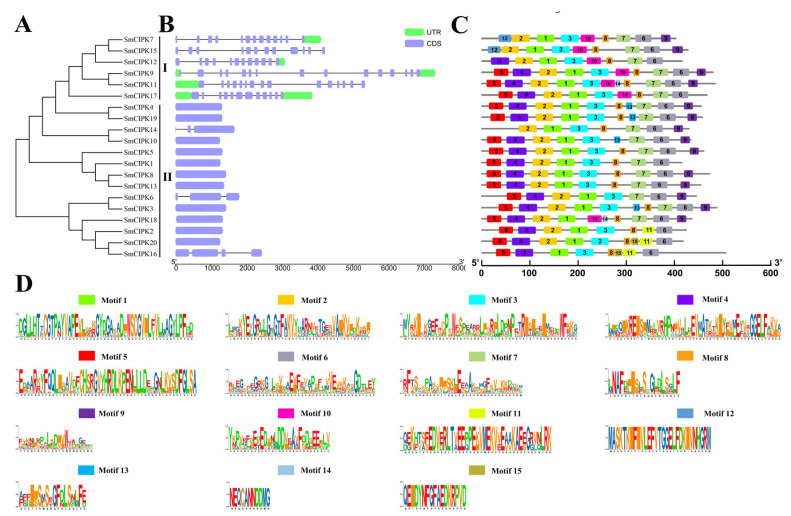
Phylogenetic relationship, gene structure and conserved motif analysis of *SmCIPKs*. (**A**). Phylogenetic tree of SmCIPKs. (**B**). Schematic diagram of intron and exon structures of SmCIPKs. Blue boxes represent exons, green boxes represent untranslated regions, and straight lines represent introns. (**C**,**D**). The different colored boxes represent different conserved motifs and specific amino acid sequences.

**Figure 3:**
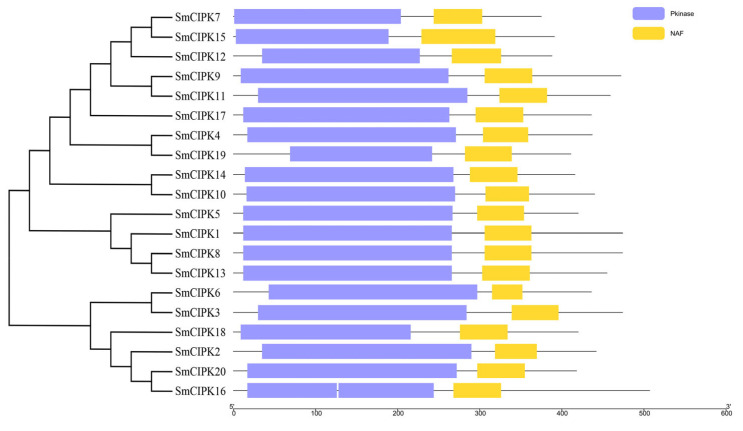
Structure of Pkinase domain or NAF domain of SmCIPKs.

Structural characterization of the *CIPK* gene family of *S. miltiorrhiza* was performed according to the order of gene arrangement in the evolutionary tree (Figure 2A). In the gene structure diagram (Figure 2B), the *SmCIPK* genes were found to be between 1.2–7.5 kb in length, with 11–14 intron numbers for the six *SmCIPKs* in group I, two introns for *SmCIPK14* and *SmCIPK6*, three intron numbers for *SmCIPK16*, and 0 intron numbers for the remaining 11 *SmCIPKs* in group II. As shown in Figure 2C, the analysis of MEME sites showed that all 6 SmCIPKs in the first group contained motif 10, and only SmCIPK18 in the second group did. SmCIPK2, SmCIPK20 and SmCIPK16 in the second group are evolutionarily related, and they all contain motif 11. Additionally, all except SmCIPK7, SmCIPK15 and SmCIPK12 contain motif 5. Therefore, these results suggest that motif 5, motif 10 and motif 11 may enrich the diversity of CIPKs in *S. miltiorrhiza*. Equally, these SmCIPKs had similar motif arrangements and motif numbers in different subgroups, indicating that the protein structures were conserved in the same subgroup, but the specific functions of the conserved motifs need to be further investigated. Undoubtedly, the conserved domain analysis showed that among the 20 SmCIPK proteins, all members had typical Pkinase and NAF domains except for SmCIPK16, whose domain Pkinase was split into two segments (Figure 3).

### 2.3. Expression Profiles of SmCIPK Genes

**Figure 4:**
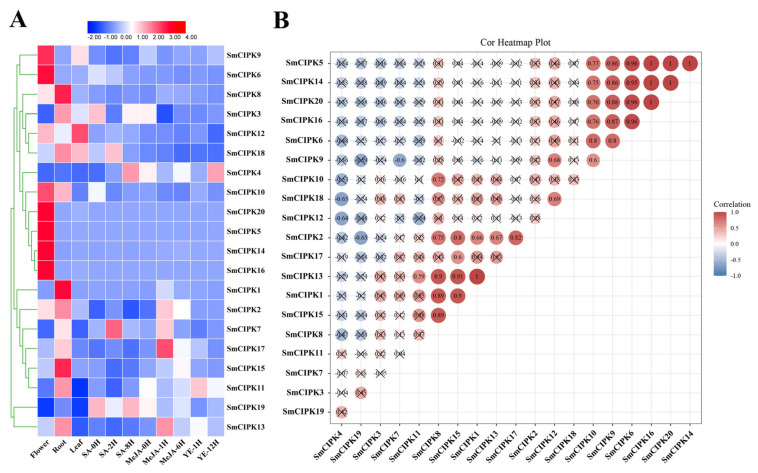
Expression pattern analysis of *SmCIPKs*. (**A**). Heat map of *SmCIPKs* expression in different tissues under different treatments. (**B**). Correlation analysis of the expression patterns between different *SmCIPKs*. Red color indicates positive correlation between two genes, blue color indicates negative correlation between two genes, and the number in each cell indicates the correlation coefficient. (Correlations > 0.5 were considered significant).

To understand the expression characteristics of *SmCIPK* genes in different tissues and under different treatment conditions, transcriptome data of three tissues of *S. miltiorrhiza* (flowers, roots, leaves, and three treatments (MeJA, SA and YE) were downloaded. The results of the heat map showed that all *SmCIPKs* were expressed more highly in flowers and roots than in leaves except for *SmCIPK12* (Figure 4A). After 2 h of SA treatment, 7 genes were up-regulated and 10 genes were down-regulated; meanwhile, after 8 h of SA treatment, their expression levels were reversed and correspondingly 7 genes were up-regulated and 10 genes were down-regulated. Of note, more than 64% of the 17 genes that changed significantly in SA treatment showed opposite trends in MeJA treatment 1 h and 6 h. For the YE treatment only 6 genes were up-regulated treatment after 1 h. Interestingly, *SmCIPK13* was highly expressed and induced by SA in all three tissues. As shown in the Figure 4B correlation heat map, SmCIPK5 and SmCIPK10 had the most members of positive correlations with 5 each. Consequently, these results suggest that *SmCIPKs* function primarily in flowers and roots and a potential crosstalk between SA, MeJA and Ca^2+^ signaling pathways. 

### 2.4. Localization of SmCIPK13 Protein and Analysis of Its Response to Abiotic Stresses

**Figure 5:**
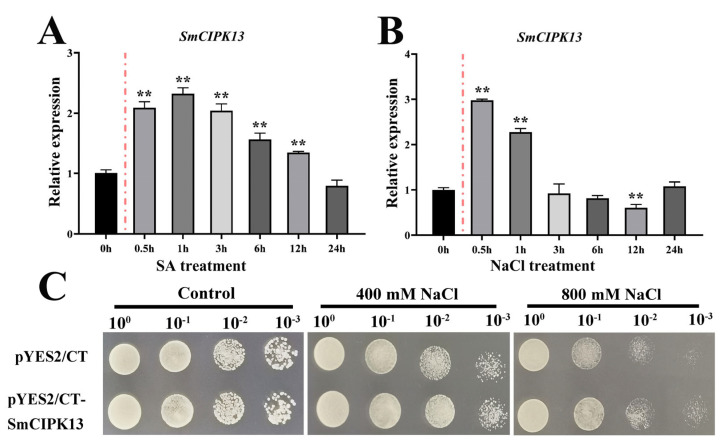
Expression analysis of *SmCIPK13* under SA and NaCl treatment and characterization of its function in yeast. (**A**,**B**). SmCIPK13 was induced at expression levels of 0, 1, 2, 3, 6, 12 and 24 h by 80 µM SA and 150 mM NaCl. (**C**). Growth of yeast that have been transformed with SmCIPK13 grown in SD/-Ura, SD/-Ura medium containing 400 mM and 800 mM NaCl. (** *p* < 0.01).

**Figure 6:**
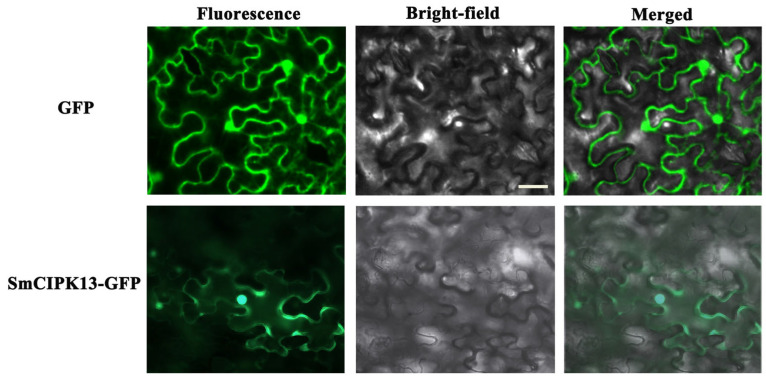
Subcellular localization of SmCIPK13 in tobacco. The scale represents 100 µm.

The results confirm prediction of SmCIPK13 localization and the hypothesis that SmCIPK13 is not only induced by SA, but also responds to NaCl. As shown in Figure 5A, after reaching a maximum at 0.5 h after 80 mM SA treatment, the transcript abundance of *SmCIPK13* showed a decreasing trend until it picked up at 24 h. After 150 mM NaCl treatment, the expression level of *SmCIPK13* was at least 2-fold higher at 1 h than at 0 h, and then continued to decline (Figure 5B). Thus, SmCIPK13 may be involved in the response of *S. miltiorrhiza* to salt stress. As shown in Figure 6, the localization of SmCIPK13 protein was essentially the same as that of the control, with distribution in the cytoplasm, cell membrane and nucleus.

### 2.5. Expression and Overexpression of SmCIPK13 Promoted Salt Resistance in Yeast and Arabidopsis, Respectively

**Figure 7:**
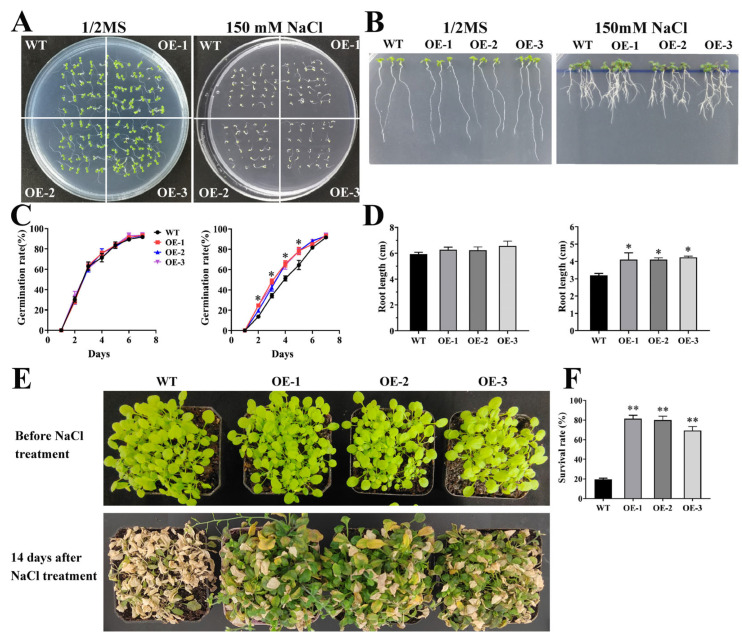
Overexpression of *SmCIPK13* enhances salt resistance in *Arabidopsis*. (**A**,**B**). Seed germination and root length elongation phenotypes of transgenic lines and WT grown in medium of 1/2 MS and 1/2 MS containing 150 mM NaCl up to day 7. (**C**,**D**) Analysis of seed germination and root length elongation of transgenic lines and WT grown in 1/2 MS and containing 150 mM NaCl in 1/2 MS medium for 7 days. (**E**,**F**) Phenotypes of three-week-old transgenic *Arabidopsis* and WT before and after two weeks of 150 mM NaCl treatment and survival after treatment (**F**). (* *p* < 0.05, ** *p* < 0.01).

To elucidate whether SmCIPK13 improves the salt resistance, transgenic *Arabidopsis thaliana* positive lines and positive transformants of yeast were obtained. As in Figure 5C, yeast that had been transformed with plasmid pYES2/CT-*SmCIPK13* grew faster on SD/-Ura medium containing 400 mM and 800 mM NaCl compared to the control (empty yeast), and the growth was the same on SD/-Ura medium. Under normal growth conditions, there was no significant difference in seed germination rate and root elongation between the transgenic lines and WT (Figure 7A,C). Notably, on 1/2 MS medium containing 150 mM NaCl, the transgenic lines had significantly higher seed germination rates than WT between days 2 and 5, while the transgenic lines also had significantly higher root lengths than WT on day 7 (Figure 7B,D). Consistently, the three-week-old transgenic lines grew as well as WT, and the survival rate of *SmCIPK13* overexpressing *Arabidopsis* was significantly higher than that of WT after two weeks of 150 mM NaCl treatment (Figure 7E,F). These results demonstrate that SmCIPK13 can enhance salt tolerance in *Arabidopsis* and yeast.

## 3. Discussion

Phylogenetic and gene structure analysis of the *S. miltiorrhiza CIPK* gene family showed that introns ranged from 0 to 14, but those classified in class I all had more than 10 introns, while those in class II had between 1 and 3 introns (Figure 2A,B). These results suggest that *SmCIPK* family gene structure introns have been lost during the evolutionary process. This is similar to the results reported for other species *Gossypium hirsutum Linn* and *Saccharum officinarum* [20,21].

CIPKs are widely distributed within cells. The reported AtCIPK1/2/3/4/7/8/14/2/24 are localized everywhere in the cell, including the cytoplasm, nucleus, plasma membrane, and endosomes [22,23,24,25]. Importantly, the subcellular localization of CBL-CIPK complexes depends on CBLs. CBLs act as Ca^2+^ receptors, and upon sensing Ca^2+^ signals, EF-hand binds Ca^2+^, its conformation changes, and then binds to the NAF domain of CIPKs, stabilizing CBL-CIPK complexes by hydrophobic interaction [26]. SmCIPK13 is predicted to localize in the cytoplasm but is found in the cytoplasm, cell membrane, and nucleus, a phenomenon that occurs because it may interact with the NtCBL protein in tobacco which influences its localization (Figure 5). 

Analysis of gene duplication, interaction and expression of the *CBL* and *CIPK* gene families revealed that differential gene expression patterns and highly specific interactions together maintain the balance of interacting CBL and CIPK proteins [27]. Our co-expression results showed that the expression patterns of one-third of *SmCIPK* genes were highly specific, and there were also cases where one *SmCIPK* was highly correlated with the expression of several other *SmCIPK* genes (Figure 4A,B). These results suggest that SmCIPK may be regulated by a single SmCBL or multiple SmCBLs, and that there is functional overlap and crosstalk between SmCIPKs, which is also consistent with the previous findings.

Previous studies have shown that in plants the CBL-CIPK signaling pathway can regulate ion homeostasis in vivo, mainly regulating the balance of Na^+^, K^+^, Mg^2+^, NO^3−^ and H^+^ [28,29,30,31,32,33,34,35]. The AtCBL4 (SOS3) (salt overly sensitive 3)-AtCIPK24 (SOS2) (salt overly sensitive 2) complex regulates sodium-potassium ion homeostasis and improves the tolerance of *Arabidopsis* to salt stress. Further studies revealed that AtCBL4-AtCIPK24 phosphorylates plasma membrane AtNHX7 (SOS1), transports excess ions out of the cell, maintains intracellular Na^+^ homeostasis, and prevents plant injury [13,28]. Under low potassium conditions, AtCBL1/AtCBL9-AtCIPK23 can phosphorylate downstream K^+^ channel protein AKT1 (*Arabidopsis* K^+^ transporter 1), which in turn activates downstream KC1 (K^+^ channel protein 1), which negatively regulates AKT1, forming a cascade reaction [31,36]. Ca^2+^ and ROS signals influence each other when they are just generated, and AtCBL1/AtCBL9 can both interact with AtCIPK26 to enhance ROS signaling [37]. AtCIPK24 regulates ion homeostasis at the vesicle and detoxifies plant cells [23]. In *Arabidopsis*, AtCIPK11/PKS5 can interact with AtCIPK24 (SOS2), phosphorylate AtCIPK24, and promote the interaction of AtCIPK24 with 14-3-3 protein, which in turn inhibits AtCIPK24 activity, and conversely, can promote the interaction of 14-3-3 protein with AtCIPK11 under salt stress, which derepresses AtCIPK11 and AtCIPK24 kinase activity [38]. Our functional characterization of *SmCIPK13* in *Arabidopsis thaliana* and yeast revealed that *SmCIPK13* enhances salt tolerance. Taken together, the function of SmCIPKs is conserved, but the downstream target genes of *SmCIPK13* and the reciprocal protein SmCBLs need to be studied more deeply. 

In recent years, with the rapid development of sequencing technology, the genomes of many plants have been published and continuously improved, and *CIPK* gene families in different species have been identified one after another. Crucially, the present study is scientifically important for the in-depth study of the regulatory networks involved in CBL-CIPK, the biological functions exercised and comparative genomic studies, and is a major inspiration for the expansion of studies to target species using the research results of model crops such as *Arabidopsis thaliana*.

## 4. Material and Methods

### 4.1. Plant Materials and Treatment

Weigh 0.5 g of hairy roots induced by *Agrobacterium tumefaciens* C58C1 in 6.7V liquid medium for three weeks starting with transfer to 6.7V liquid medium containing 80 µM SA and 150 mM NaCl. Samples were collected after 0, 0.5, 1, 2, 3, 6, 12 and 24 h of treatment and then stored at −80 °C until RNA extraction; untreated hairy roots were used as controls. The extracted RNA was reverse transcribed into cDNA according to the instructions of the HiScript III 1st Strand cDNA Synthesis Kit (Vazyme Biotech, Nanjing, China). The qPCR method uses the steps that have been reported [39]. Each sample consisted of three biological replicates.

### 4.2. Identification and Characterization of Gene Family Members

To identify members of the CIPK family in *S. miltiorrhiza*, the following three steps were taken. First, the HMM models of Pkinase (PF00069) and NAF (PF03822) were downloaded from the Pfam database (http://pfam.xfam.org/, accessed on 5 August 2021), and through the software HMMER3 [40] (http://www.hmmer.org, accessed on 5 April 2022) to search the protein database (E-value < 0.01), respectively. Second, using the *Arabidopsis thaliana* CIPK family protein sequences as the query, we set the E value to 1 × 10^−10^ using ncbi-blast+ v2.8.1 software, compared to the protein database, and counted the results with identity greater than or equal to 50%. Third, combining the results of the above two steps, the non-redundant sequences were uploaded to the Pfam database, and the SMART database (http://smart.embl-heidelberg.de/, accessed on 5 August 2021) was further analyzed to determine whether CIPK contains conserved domains. Ip (isoelectric point) and MW (molecular weight) analysis were performed using the online website (http://web.expasy.org/protparam/, accessed on 14 October 2021) and subcellular localization prediction was accomplished by http://www.csbio.sjtu.edu.cn/bioinf/Cell-PLoc-2/, accessed on 14 October 2021. The amino acid sequences and nucleic acid sequences of SmCIPKs are in Supplementary File S1.

### 4.3. Protein Sequence, Phylogenetic Tree Construction and Gene Structure Analysis

Sequence analysis included gene structure analysis, conserved motif analysis, conserved structural domain analysis and multiple sequence alignment analysis. Gene structure analysis was performed using CDS sequences and gene sequences, and mapping was analyzed using the online website GSDS2.0 (http://gsds.gao-lab.org/, accessed on 12 August 2021) [41]. Conserved motif analysis was performed using protein sequences and analyzed using the online website MEME suite 5.0.4 (https://meme-suite.org/meme/, accessed on 12 August 2021) [42], with motif numbers adjusted until the number of motifs of members differed, with a final selected number of 15.

Conserved domains were analyzed using the website pfam search (http://pfam.xfam.org/search, accessed on 10 August 2021) to filter the most reliable results. To better display motif features, motif redrawing was performed using the Redraw Motif Pattern function of TBtools [43]. Multiple sequence alignment analysis was performed using the software DNAMAN 10, using default parameters.

Using muscle alignment multiple sequences in the software MEGA7 [44], evolutionary trees were constructed using the Neighbor-Joining method (NJ), selecting parameters poisson model, pairwise deletion, bootstrap (self-expansion value) of 1000, and validating using Maximum Likelihood (ML) method; otherwise, default parameters were used, and subsequent tree beautification was performed using software Adobe Illustrator CS6.

### 4.4. Analysis of SmCIPKs Expression Patterns in Different Tissues and under Different Treatments

Transcriptome data for three treatments (SA, MeJA and yeast extract) and three tissues (leaves, flowers and roots) are available for download at www.ncbi.nlm.nih.gov/sra, accessed on 21 December 2021 under the accession numbers SRR1043998, SRR1045051, SRR1020591, SRX1423774, SRX2992229 SRX2992230, SRX2992231, SRX2992232, and SRX2992233. The heat map was prepared using the software TBtools [44]. The FPKM values of *SmCIPK* involved in the heat map are shown in Supplementary Table S1.

### 4.5. Plasmid Construction, Transgenic Arabidopsis and Stress Treatment

The plasmid construction method was performed according to the instructions of the ClonExpress^®^ MultiS One Step Cloning Kit (Vazyme Biotech, Nanjing, China). All primers involved in the above experiments are shown in Supplementary Table S2. For vector construction for using in transgenic *Arabidopsis*, the full-length ORF (open reading frame) of *SmCIPK13* was PCR amplified and constructed into the pCAMBIA1304-GFP vector. For resistance experiments in yeast, the full-length CDS (coding sequence) of SmCIPK13 was constructed into the pYES2/CT vector. 

T3 generation homozygous transgenic *Arabidopsis* lines were obtained by plate screening on 1/2 MS medium containing 50 mg/mL *hygromycin* and detection at the molecular level by PCR [45]. For the seedling phenotyping experiment under salt stress, transgenic lines (OE-1, OE-2, and OE-3) and WT (wild type) were grown to the fourth week (24 °C, 16 h/8 h, light/dark). Subsequently, the phenotype was observed and the survival rate was calculated after 150 mM NaCl treatment for two weeks.

For experiments on the effect of salt stress on seed germination rate, sterilized seeds of transgenic lines and WT were germinated on 1/2 MS (control) and 1/2 MS containing 150 mM NaCl, individually, and germination rate was observed and counted for 7 days. For the experiment on the effect of salt stress on root elongation, sterilized seeds of transgenic lines and WT were germinated on 1/2 MS and left upside down for two days, and then plants of equal growth were selected and transferred to 1/2 MS medium with and without 150 mM NaCl, respectively, while root length growth was measured up to day 7 of growth.

### 4.6. Subcellular Localization of SmCIPK13 and Salt Stress Tolerance Assay in Yeast

To determine the subcellular localization of SmCIPK13, we transformed pCAMBIA1304-*SmCIPK13*-GFP into *Agrobacterium tumefaciens* (pSoup-p19) and injected tobacco according to the previous method [46]. The tobacco was grown in the dark for one day and then incubated in the light for two days before being observed under a confocal microscope. Expression of the empty vector in tobacco was used as a control. 

To initially investigate the function of SmCIPK13 in abiotic stresses, the pYES2/CT-*SmCIPK13* was transformed in *Saccharomyces cerevisiae* (INVSc1). Positive transformants were induced at 20 mM galactose for 24 h in YPDA liquid medium, diluted to 10^0^, 10^−1^, 10^−2^ and 10^−3^-fold and then 10 µL were transferred on SD/-Ura medium containing 400 and 800 mM NaCl and then photographed.

### 4.7. Statistics Analysis

Whether the data were significant or not was analyzed using SPASS 26.0 for one-way ANOVA and GraphPad Prism 8.0 for graphing, and the data were obtained based on three biological replicates and three technical replicates. The correlation was analyzed using the Pearson correlation coefficient approach.

## Figures and Tables

**Table 1:** Physiological characterization of *SmCIPKs* genes.

Gene Name	Gene ID	CDS	AA	pI	Mw (Da)	Subcellular Localization
*SmCIPK1*	SMil_00000703	1263	420	9.29	47,270.22	Cytoplasm
*SmCIPK2*	SMil_00000704	1329	442	8.81	49,427.94	Cytoplasm
*SmCIPK3*	SMil_00000853	1425	474	8.56	53,056.17	Cytoplasm
*SmCIPK4*	SMil_00001122	1311	436	9.28	49,667.43	Cytoplasm
*SmCIPK5*	SMil_00003013	1323	440	9.02	49,944.58	Cytoplasm Nucleus
*SmCIPK6*	SMil_00003083	1311	436	9.25	49,042.18	Cytoplasm
*SmCIPK7*	SMil_00003987	1128	375	5.99	42,844.14	Cytoplasm
*SmCIPK8*	SMil_00004681	1425	474	8.88	53,641.64	Cytoplasm Nucleus
*SmCIPK9*	SMil_00008243	1380	459	8.97	51,920.95	Centrosome Cytoplasm Nucleus
*SmCIPK10*	SMil_00009044	1251	416	9.07	46,960.19	Cytoplasm
*SmCIPK11*	SMil_00010169	1419	472	5.69	54,055.99	Centrosome Cytoplasm Nucleus
*SmCIPK12*	SMil_00012715	1167	388	8.15	43,862.71	Cytoplasm
*SmCIPK13*	SMil_00014175	1368	455	9.14	51,091.17	Cytoplasm
*SmCIPK14*	SMil_00021346	1236	411	6.51	46,210.19	Cytoplasm
*SmCIPK15*	SMil_00025018	1176	391	5.52	44,490.61	Cytoplasm
*SmCIPK16*	SMil_00025262	1524	507	9.56	57,920.08	Centrosome Cytoplasm Nucleus
*SmCIPK17*	SMil_00026858	1380	459	6.01	51,419.66	Cytoplasm
*SmCIPK18*	SMil_00026916	1263	193	6.21	47,926.96	Cytoplasm
*SmCIPK19*	SMil_00027660	1314	437	9.04	49,288.96	Cytoplasm
*SmCIPK20*	SMil_00028555	1257	418	8.92	47,519.09	Cytoplasm

## Data Availability

Data is contained within the article or Supplementary Materials.

## Supplementary Materials

The following are available online at https://www.mdpi.com/article/10.3390/ijms23126861/s1.
